# To ski or not to ski? A meta-analysis of more than 750,000 upper extremity injuries comparing skiing and snowboarding

**DOI:** 10.1177/17585732251326905

**Published:** 2025-04-22

**Authors:** Aline Chauffard, Aurélien Traverso, Gwenaël Kaminski, Jolanda Elmers, Olivier Borens, Frédéric Vauclair

**Affiliations:** 1 Department of Orthopaedics and Traumatology, Centre hospitalier universitaire vaudois (CHUV), Lausanne, Vaud, Switzerland; 2 UMR 5263, Laboratoire CLLE-LTC, Toulouse, France; 3 Centre Hospitalier Universitaire Vaudois, Faculty of Biology and Medicine Library, Lausanne University Hospital and University of Lausanne, Lausanne, Switzerland; 4 282013 Department of Bone and motion, Clinique Bois-Cerf, Lausanne, Switzerland

**Keywords:** traumatic injuries, upper extremity, winter sports, ski, snowboard

## Abstract

**Background:**

Alpine skiing has seen advancements in equipment since the year 2000, with the appearance of ski carving. Its impact on upper extremity injuries has yet to be proven. We conducted a meta-analysis to determine the epidemiology of upper extremity injuries in alpine skiing and snowboarding, its chronological evolution in the last two decades, and the impact of carving.

**Method:**

A systematic search in PubMed was conducted including studies from 1939 to 2024. The search strategy used text words and relevant indexing to identify articles discussing upper extremity injuries associated with those activities while providing statistical and epidemiological data.

**Results:**

77 studies including a total of 764,423 patients were analysed. The most commonly injured upper extremity segments are the shoulder (37%) for skiing and the wrist (36%) for snowboarding. The main upper extremity dislocation is glenohumeral (36%) for skiing and the elbow (46%) for snowboarding. Hand injuries are significantly more prevalent while skiing, but the rest of the upper extremity injuries are significantly more prevalent with snowboarding.

**Discussion:**

The time trends from the year 2000 and on have shown a significant increase in upper extremity injuries with ski carving. Snowboarding injury epidemiology has not significantly changed in this period.

## Introduction

Musculoskeletal injuries are frequently associated with winter sports. Usually, alpine skiing and snowboarding accidents cause lower extremity injuries, with knee injuries accounting for 30–50% of all accidents.^1,2^

Skiing was the main sport on the slopes for a long time, but snowboarding has gradually become more and more popular. The early prototypes of snowboarding date back to the 1960s, but the real introduction of this sport began in the 1970s in the USA.^3,4^ However, snowboarders were at first not always welcome in ski resorts and had to wait until the 1990s to be universally accepted.^3^ Therefore, publications prior to the 1990s did not integrate snowboarding in their data and since this time period, the wrist is considered the most common location for injuries while snowboarding.^5^

A revolution in winter sports was the development of carving skis. Carving is a technique in which the middle of the ski is slimmer than the front and back, making it easier and more efficient to turn.^6^ It was first developed in 1993 by the Slovenian brand *Elan,*^7^ but only began to be globally commercialized by brands in the 2000s. This new ski design has modified the epidemiology of ski-related injuries, and it is now accepted that carving represents a risk factor for internal knee injuries, especially anterior cruciate ligament (ACL) tears.^8,9^

While ski material evolution has proven to have a causal effect on lower extremity injuries,^8,9^ its effect on upper extremity injuries has not been evaluated in detail yet. To the best of our knowledge, there are no published systematic reviews addressing upper extremity ski-related injuries to date.

The primary outcome was to confirm the hypothesis that snowboarding would cause more upper extremity injuries than alpine skiing. The secondary outcomes were to describe the epidemiology of upper extremity injuries in both ski and snowboard, determine the impact of carving on upper extremity injuries, and determine if the rate and severity of upper extremity traumatic injuries in snowboard would remain more consistent due to the lake of significant technical changes.

## Materials and methods

### Literature search and inclusion/exclusion criteria

**Figure 1.**
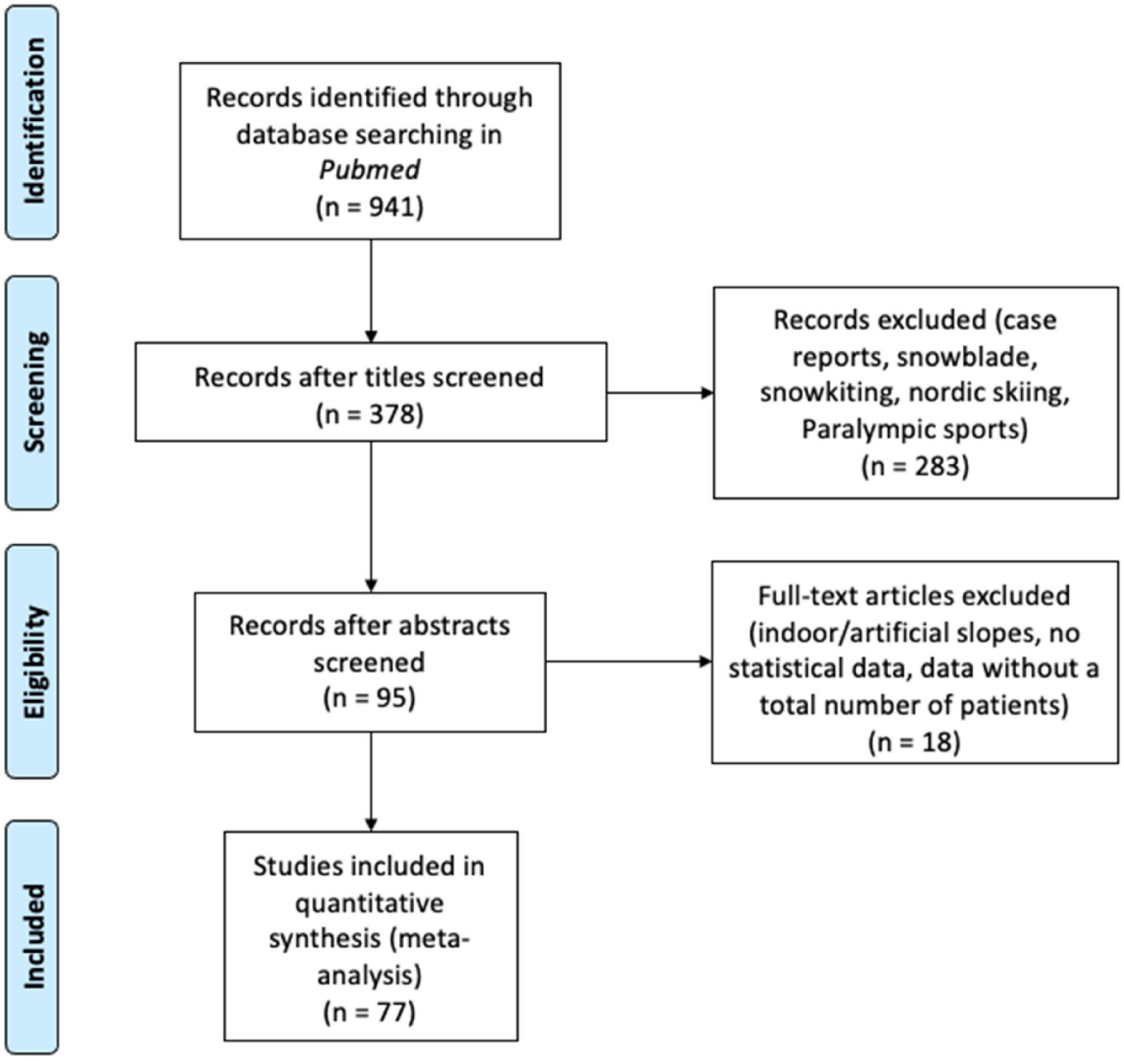
Flow diagram adapted from PRISMA.

A systematic search in PubMed using the PRISMA Figure 1 process for the bibliographic review^10^ was conducted independently by two authors (AC and AT) according to the inclusion criteria. Searches with PubMed were last conducted in June 2024.

The principal keywords used in these databases were “snowboard,” “skiing,” all anatomical entities of the upper extremity (bones, joints, muscles, and ligaments), and all types of injuries possible. The complete description of the search strategy is available in the supplementary information (see Electronic Supplementary Material).

We included both prospective and retrospective studies that conducted their own data collection. Articles focusing on upper extremity injuries associated with alpine ski, snowboard, and telemarking were included. For articles discussing more than one of these sports, the statistical data had to be explicitly provided in distinct data sets to avoid conflation.

Other winter sport disciplines such as snowblading, snowkiting, and indoor or artificial slope involvements as well as Paralympic forms of these activities were excluded. Another exclusion criterion was incomplete data for statistical analysis (for example, a lack of total patient number or lack of prevalence. Case reports were also excluded.

With these criteria considered, all articles retrieved using our search strategy in Pubmed were subsequently reviewed in detail. The majority of articles could be eliminated solely on the title, whereas others were excluded upon reviewing the abstract or even the full text.

### Review criteria

Our literature review was based on 77 studies with a total of 764,423 patients who met the inclusion criteria. Characteristics of the included studies such as first author, year of publication, design, number of patients by sport type, gender prevalence, and age are summarized in Table 1. Over the period from 1939 to 2024, a total of 77 studies. The mean age of patients was 26.1 years old, and there was a male prevalence of 0.67.

**Table 1.** List of studies used in the systematic review (Snow = snowboard, Tele = telemark, P = prospective, R = retrospective, TPN = total patient number, ? = unknown).

Author	Publication year	Study design	TPN			Male prevalence	Mean age
			Ski	Snow	Tele		
Abu-Laban^11^	1991	P	697	132			
Aslam and Thomas^12^	2004	P	16	27		0.83	28
Bachmann et al.^13^	2008	R	31			0.72	22
Basques et al.^14^	2016	R	3351	2704		0.75	31
Bissell et al.^15^	2008	P	18,692	2260			35
Bladin et al.^16^	1993	P	450	276		0.74	21
Blitzer et al.^17^	1984	R	3182				
Callé and Evans^18^	1995	R		1052		0.77	19
Carr et al.^19^	1981	P	1711			0.57	24
Cattermole^20^	1999	R	59				
Chow et al.^21^	1996	P	478	390		0.81	20
Corra et al.^22^	2004	R	1003	331		0.58	32
Coury et al.^5^	2013	P	1196	397		0.54	32
Davidson and Laliotis^23^	1996	R	8046	929		0.5	29
Davidson and Laliotis^24^	1996	R	24,340			0.5	28
De Roulet et al.^25^	2017	R	1353	1216		0.82	
Deibert et al.^26^	1998	P	10,113				
Dickson et al.^27^	2011	P	802			0.57	22
Dohjima et al.^28^	2001	R	5048	2552			23
Drkulec and Letts^29^	2001	R		118		0.82	14
Ehrnthaller et al.^30^	2015	R		341		0.65	26
Ekeland et al.^31^	2018	R	3569	1236			
Emery et al.^32^	2006	R		142		0.52	
Federiuk and Mann^33^	1999	R			494	0.74	43
Felkai et al.^34^	2017	R	201	19			
Gallo-Vallejo et al.^35^	2017	R	28	11		0.6	
Ganong et al.^36^	1992	P		424		0.74	20
Geyer and Beyer^37^	1989	P	414				
Gigli Berzolari et al.^38^	2009	R	1264	228		0.58	35
Hagel et al.^39^	2005	P		1108		0.69	
Hagel et al.^40^	1999	P		557			
Hisdal et al.^1^	2017	R	2130	1051		0.6	
Hurt et al^41^	2022	R	140,362	182,201			
Idzikowski et al.^42^	2000	P		7430		0.74	
Ishimaru et al.^43^	2012	P		5561		0.68	
Johansen et al.^44^	2015	R			149	0.63	
Khalilifar et al.^45^	2012	P	1167			0.75	28
Kim et al.^46^	2012	P	9465	2260		0.58	28
Kocher and Feagin^47^	1996	R	3451			0.75	35
Köhne et al.^48^	2005	P	2433	1414		0.59	29
Kuriyama et al.^49^	1984	R	14,952			0.65	
Machold et al.^50^	2000	R		152			
Made and Elmqvist^51^	2004	P		568		0.66	19
Major et al.^52^	2014	R		574			
Matsumoto et al.^53^	2004	P		5110		0.66	23
Matsumoto et al.^54^	2002	P	2175	6837		0.75	24
Oberthaler et al.^55^	1995	R		437		0.71	21
Ogawa et al.^56^	2011	R	1272	7793		0.87	28
Ogawa et al.^57^	2010	P		18,791		0.68	24
Patrick et al.^58^	2015	P		65			
Pechlaner et al.^59^	1987	R	17,999				
Pierpoint et al.^60^	2020	R	3961	2428		0.61	30
Pino and Colville.^61^	1989	R		110		0.9	21
Ruedl et al.^62^	2014	P	5860	1025		0.49	35
Russel et al.^63^	2013	R		360			
Rust et al.^64^	2013	R	821	280			
Sahlin^65^	1989	P	347			0.67	21
Sasaki et al.^66,67^	1999	R	10,152	1445			
Schrank et al.^68^	1999	R		195		0.67	22
Selig et al.^69^	2012	R	749	117		0.62	11
Sherry^70^	1984	P	1850				
Steinbrück et al.^71^	1999	P	4632	155			
Stenroos and Handoli^72^	2015	R	1991	893			
Subasi and Gur^73^	2023	R	?	?	?	?	?
Sulheim et al.^74^	2011	P	1598	1387	179	0.6	
Sutherland et al.^75^	1996	R		757			
Tapper^76^	1978	P	10,120				
Torjussen and Bahr^77^	2006	R		135		0.46	23
Torjussen and Bahr^78^	2005	P + R		98			
Tuggy and Ong^79^	2000	R			178	0.7	38
Ueland and Kopjar^80^	1998	P	2577	505	404	0.67	22
Ungerholm et al.^81^	1983	P	2756				
Warme et al.^82^	1995	P	9749	47			
Wasden et al.^83^	2009	P	989	421		0.75	36
Xiang et al.^84^	2005	P	77,300	62,000		0.66	
Yamauchi et al.^85^	2010	R		16,564		0.51	24
Zollinger et al.^86^	1994	P	?	?	?	?	?

*Accuracy level*. Due to variations in the accuracy of anatomical localization and injury type across publications, the data was hierarchized. The data was divided into two levels: high accuracy and low accuracy. The more accurate it was, the more in-depth statistical analysis was performed. When the segment or type of injury was not mentioned (meaning it was described as “upper extremity”), it was considered as “unspecified”. The low-accuracy data was only used in the descriptive analysis.

*Segment*. Due to the lack of a common definition for anatomical categories, with some studies using a joint-based classification and others a segment-based one, a standardized classification incorporating both joints and segments was used. The review focused on seven segments of the upper extremity: Shoulder girdle, Shoulder, Upper arm, Elbow, Forearm, Wrist, and Hand.

*Injury*. We gathered the injuries in large groups which enabled the cross-sectional juxtaposition of data. The three main groups of injuries were: Fracture, Dislocation, and Sprain or strain.

*Period*. In order to correlate the epidemiology with the ski material being used (carving vs. non-carving), the data was analyzed separately depending on the period of the studies. Since carving was truly introduced to the slopes in the 2000s, it is arbitrarily considered that the time before the year 2000 as a non-carving period, from 2000 to 2010 as a transition period, and after 2010 as purely a carving period. Because some data sets overlapped with these carving-related periods, the median year of each set was used as a new variable for a standardized normal distribution. This way, a prevalence was calculated for every year, which rendered comparison between periods possible. The transition period was then subdivided into 5-year phases to be more accurate. This way we were able to analyze the epidemiologic changes in the last decades and see if there was any significance between them.

*Sport*. This systematic review focused on three sports: skiing, snowboarding, and telemark. Telemark injuries were included in the study, but analyzed separately from “regular” skiing, because of the difference in bindings and technique. Our review included a total of 417,110 ski injuries, 345,909 snowboard injuries, and 1404 telemark injuries. Each sport injuries were analyzed independently to allow extraction of its data and also to permit comparisons with each other.

### Statistical analysis

*Segment datasets*. For each segment (with high accuracy), we collected information from 10 to 26 studies (out of a total of 77 surveyed). For each study, we collect the following data: number of patients sampled, number of occurrences, type of injury, sport, and period. From each study, we create a sub-base containing as many rows as individuals sampled and an “event” column, coded in a binary way: 0 meaning no lesion and 1 meaning that the individual has a lesion. Data on type of injury (three modalities), sport (three modalities), and period (five modalities) are split into separate columns. Next, we merge all the sub-bases, to finally obtain one base for each segment.

*Data analysis*. All statistical analyses were conducted with R version 4.2.0. The injury's prevalence was analyzed with generalized linear mixed models (GLMM). Study ID was set as a random effect, the injury's prevalence as the dependent variable, and sport type was considered as a fixed effect. We have built a model for each type of injury and for each segment. The same analysis is used to test the effect of the period. From our models, we computed the odds ratios (with 95% confidence intervals) to compare the relative odds of the occurrence of the outcome of interest (e.g., with the sport type variable we can compare skiing versus snowboarding). An odd ratio significantly different from 1 means that there is a significant effect of the comparison studied.

## Results

### Epidemiology

**Figure 2.**
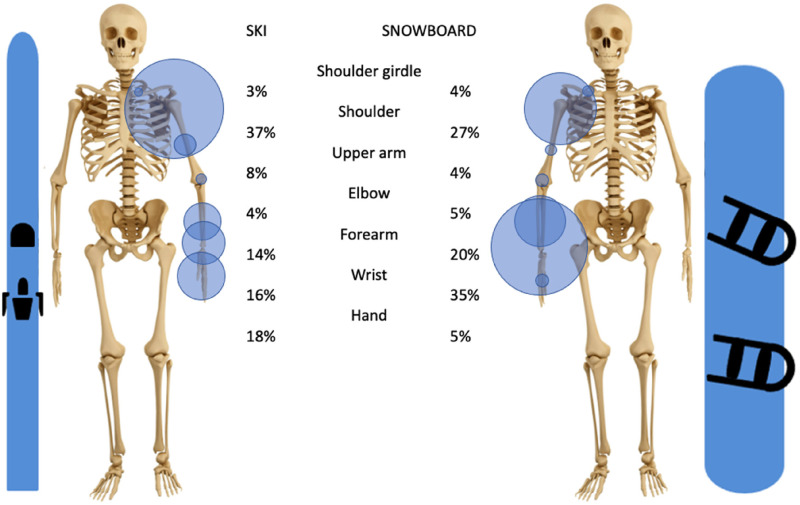
Distribution of all types of injuries of the upper extremity in skiing and snowboarding of articles used with in parenthesis the total number of patients used).

The upper limb represents 18% of all skiing injuries and 26% of all snowboarding injuries. In skiing, the three most frequently injured segments of the upper extremity are the shoulder (37% of all upper extremity injuries), the hand (18%), and the wrist (16%). In snowboarding the three most prevalent injuries are of the wrist (35%), the shoulder (27%), and the forearm (20%), Figure 2.

### Fractures

**Figure 3.**
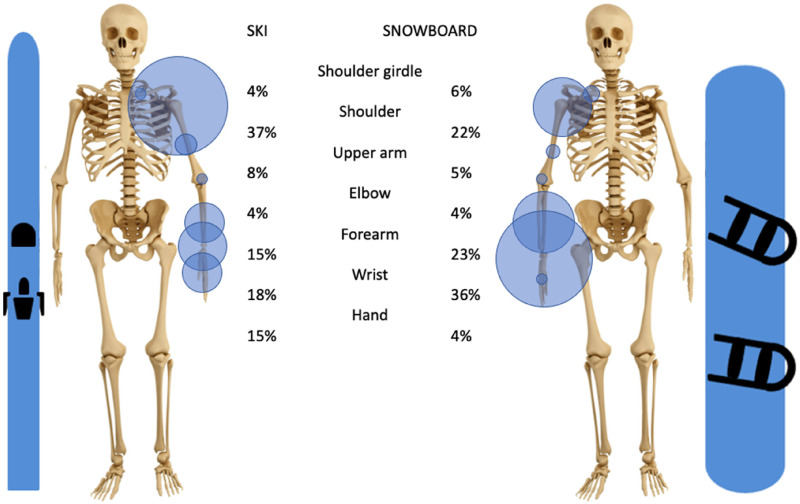
Distribution of upper extremity fractures in skiing and snowboarding h in parenthesis the total number of patients used).

**Figure 4.**
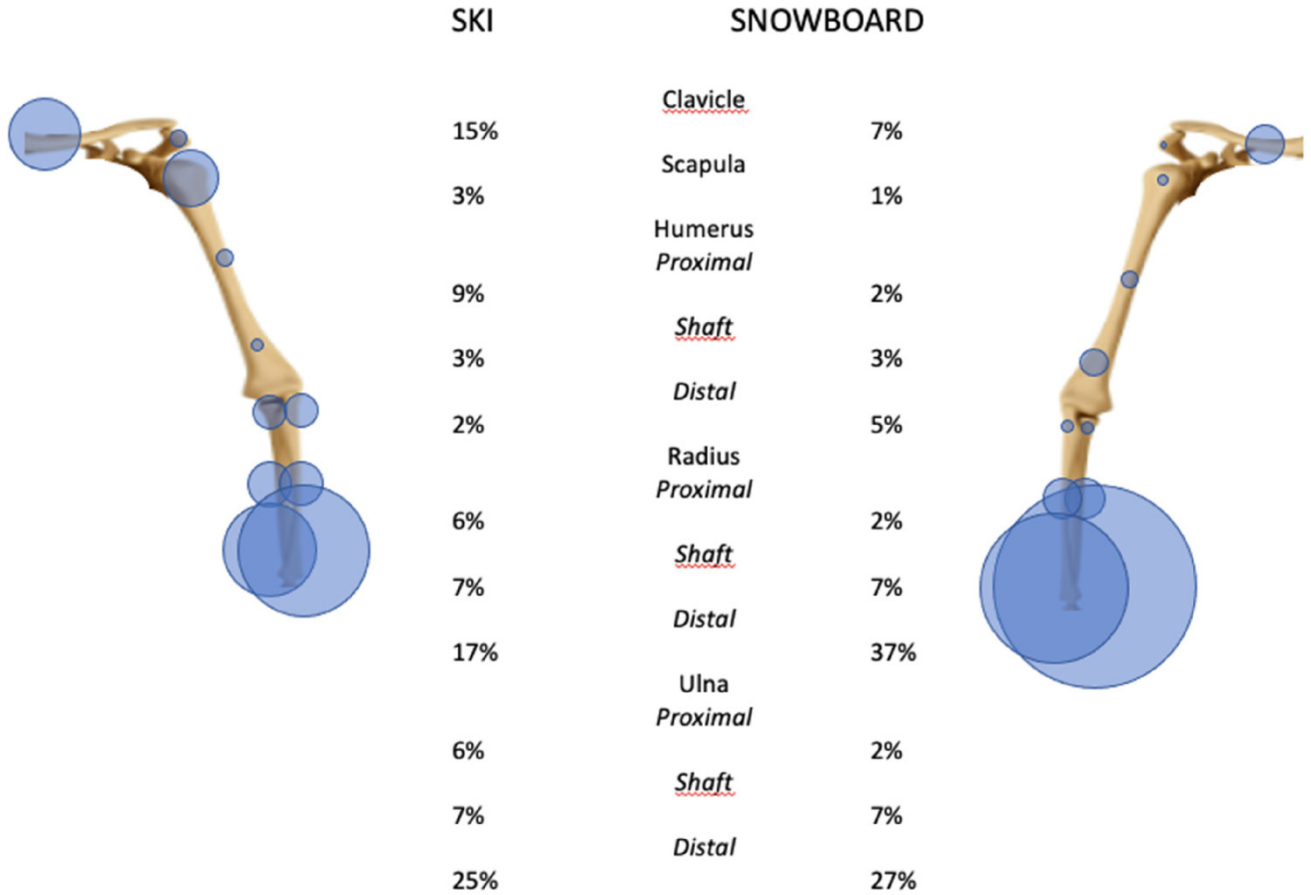
Distribution of fractures from the shoulder girdle to the forearm in skiing and snowboarding (the carp, the metacarpals, and the phalanges are not represented here).

In skiing, 5% of all injuries are fractures of the upper extremity. The three most commonly fractured bones are the humerus, the ulna, and the radius. While the humerus is mostly fractured proximally, the radius and ulna are mostly fractured distally (wrist), Figures 3 and 4.

In snowboarding, approximately 8% of all injuries are upper limb fractures. The most common fracture location is the wrist (36% of all upper extremity fractures), followed by the forearm (23%) and the shoulder (22%). Therefore, the two most fractured bones of the upper extremity are the ulna and the radius with a predomination of the distal aspect of the bone, Figures 3 and 4.

### Dislocations

**Figure 5.**
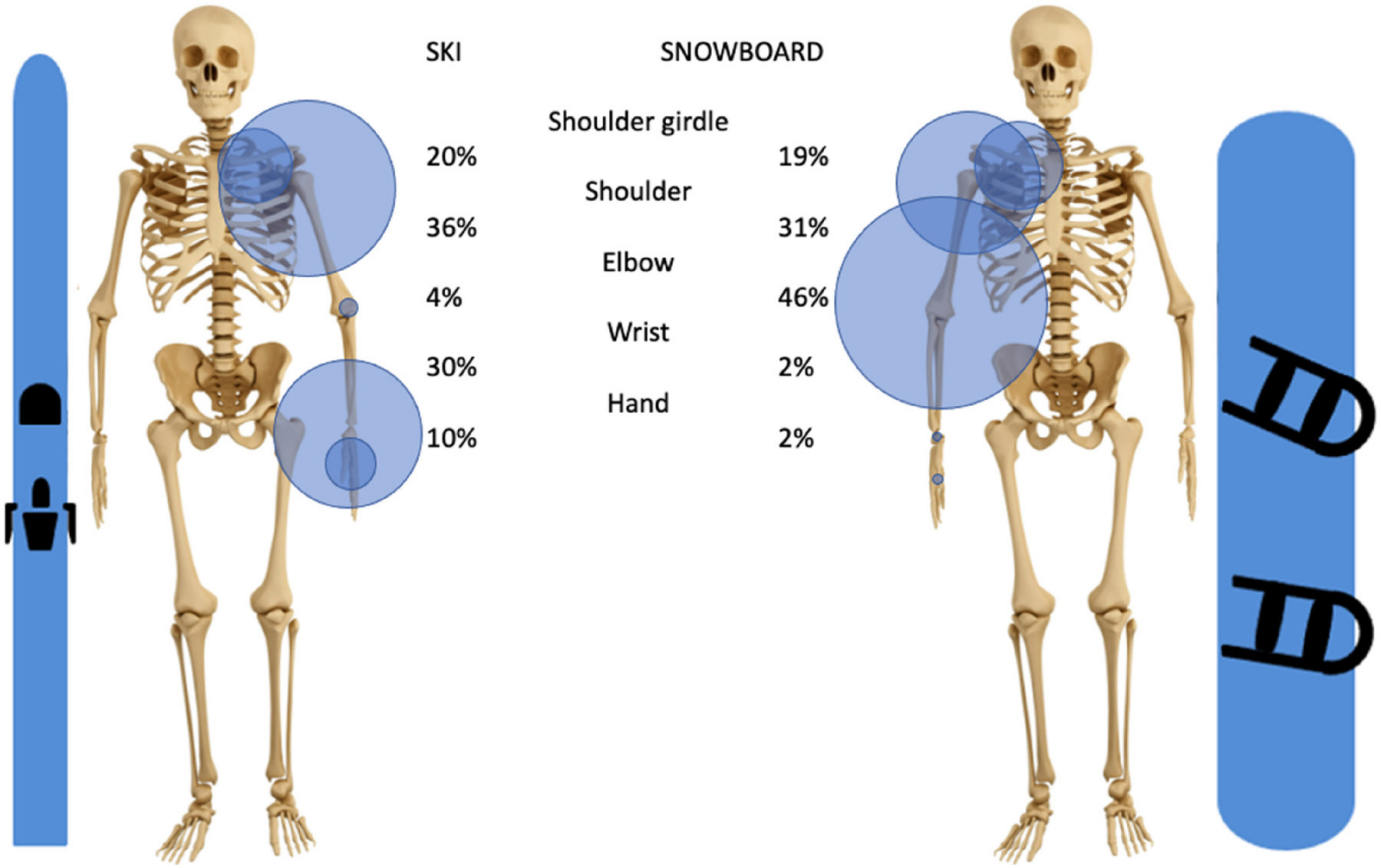
Distribution of upper extremity dislocations in skiing and snowboarding.

Dislocations affecting the upper extremity represent 2% of all skiing injuries, with glenohumeral dislocation being the main one (36% of upper extremity dislocations). In comparison, 4% of all snowboard injuries are dislocations of the upper extremity, and unlike skiing, the most common site of dislocation is the elbow (46% of all upper extremity dislocations), then the glenohumeral joint (31%) and the shoulder girdle (19%). In both sports, the shoulder girdle dislocations are almost always localized at the acromioclavicular articulation, with the sternoclavicular articulation being rarely injured (<1% in skiing and 0% in snowboarding), Figure 5.

### Ski vs. snowboard

**Figure 6.**
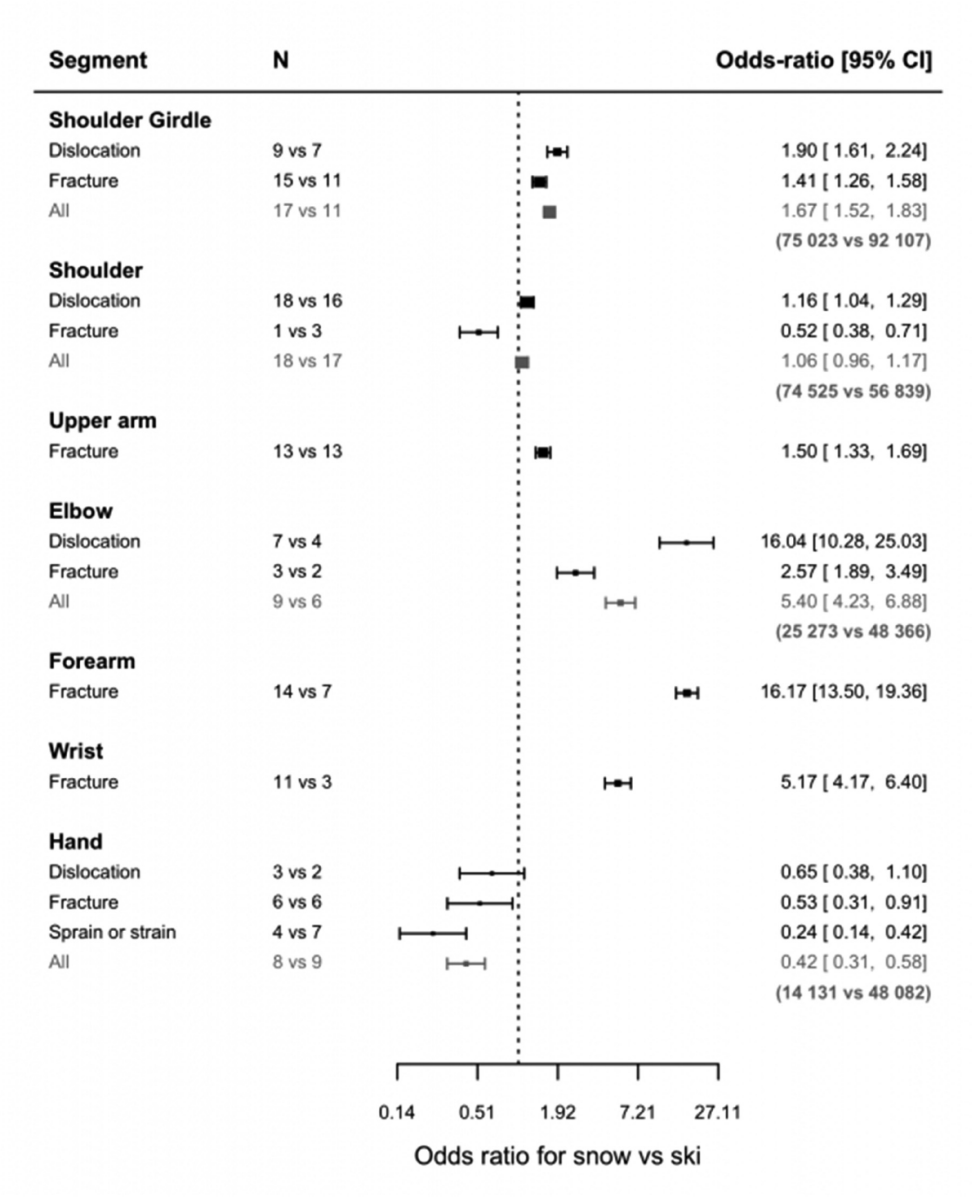
Odds ratio for snowboarding vs. skiing (*N* = number of articles used in the comparison).

Our analysis shows the effect of sport type (skiing *versus* snowboarding) on the risk of injury for six of the seven upper extremities studied (Figure 6). When comparing upper limb injuries in skiing and snowboarding, a significant difference can be noted (50 studies for skiing injuries versus 58 studies for snowboarding injuries). With snowboarding it is more likely to get a shoulder girdle (OR: 1.67 [1.52, 1.83]), upper arm (OR: 1.50 [1.33, 1.69]), elbow (OR: 5.40 [4.23, 6.88]), forearm (OR: 16.17 [13.50, 19.36]), or wrist injury (OR: 5.17 [4.17, 6.40]). However, it is less likely to get a hand injury (OR: 0.42 [0.31, 0.58]). The risk of getting a shoulder injury is not statistically different between the two sports in our sample data.

The incidence of fractures can be estimated for all seven segments. This incidence is significantly higher in snowboarding for shoulder girdle (OR: 1.41 [1.26, 1.58]), humeral diaphysis fractures (OR: 1.50 [1.33, 1.69]), fractures around the elbow (OR: 2.57 [1.89, 3.49]), forearm fractures (OR: 16.17 [13.50, 19.36]), and wrist fractures (OR: 5.17 [4.17, 6.40]), but it is more likely to get a proximal humerus (OR: 0.52 [0.38, 0.71]) or hand fracture (OR: 0.53 [0.31, 0.91]) while skiing.

*The incidence of dislocation can be estimated for four out of seven segments.* The incidence of dislocations is significantly higher in snowboarding for shoulder girdle (OR 1.90 [1.61, 2.24]), glenohumeral (OR: 1.16 [1.04, 1.29]), and elbow dislocations (OR: 16.04 [10.28, 25.03]). Hand dislocations seem to be more prevalent with skiing, yet this is not found to be significant.

### Ski vs. telemark

**Figure 7.**
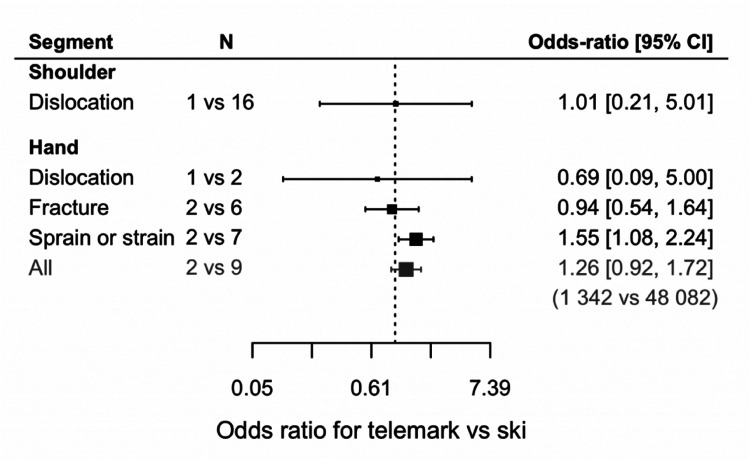
Odds ratio for telemark vs. ski (*N* = number of articles used with in parenthesis the total number of patients used).

Five studies provided insight into telemark upper extremity injuries, but only shoulder and hand injuries were reported. For these two segments, the comparison with skiing showed no significant difference (Figure 7), except for sprain or strain injuries of the hand being more prevalent in telemark. Shoulder dislocations due to telemark have a prevalence of 3.4% of all injuries, while prevalence is 3.3% with skiing. In telemark, the prevalence is as follows: hand dislocation 0.6% of all injuries (ski 0.8%), hand fracture 2.9% (ski 1.7%), and hand sprain and strain 10.8% (ski 5.1%).

### Time trends in the last two decades

**Figure 8.**
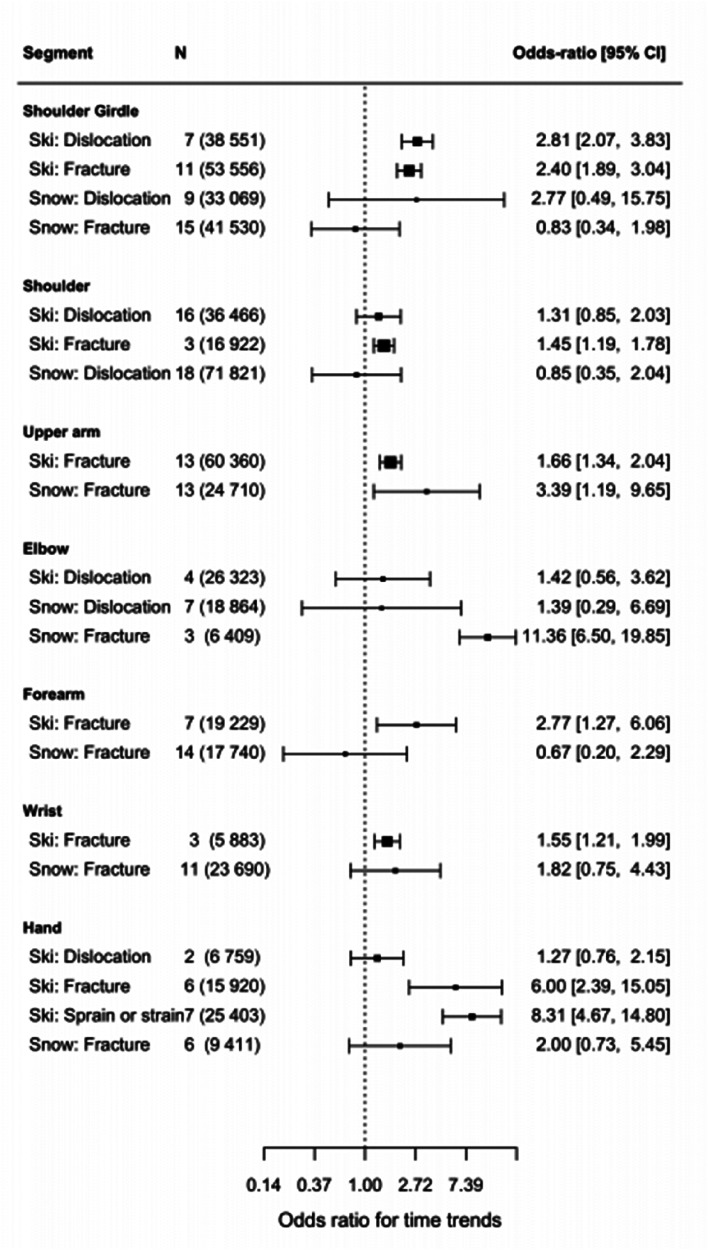
Odds ratio for time trends (*N* = number of articles used with in parenthesis the total number of patients used).

The evolution of skiing practice, in which the appearance of carving had a significant impact on six of the seven upper extremities studied (Figure 8). There is a significant time effect between shoulder girdle injuries and the introduction of carving with both more fractures (OR: 2.40 [1.89, 3.04]) and more dislocation (OR: 2.81 [2.07, 3.83]). Carving shows a significant impact on shoulder injuries for fractures (OR: 1.45 [1.19, 1.78]). There is also a slight increase (insignificant) for dislocation (OR: 1.31 [0.85, 2.03]). The same trends are found for the upper arm (OR: 1.66 [1.34, 2.04]), forearm (OR: 2.77 [1.27, 6.06]), and wrist fractures (OR: 1.55 [1.21, 1.99]) which have significantly increased with carving. There is a significant time effect for hand injuries and the introduction of carving. While dislocations did not increase significantly, both fractures (OR: 6.00 [2.39, 15.05]) and sprain or strain, which includes the skier's thumb, increased significantly (OR: 8.31 [4.67, 14.80]). There is no time effect on *elbow injuries* in carving.

Through the last decades, upper arm fractures have significantly increased for snowboarding, (OR: 3.39 [1.19, 9.65]). The elbow injuries incidence has increased, without significance for dislocation, yet significantly for fractures (OR: 11.36 [6.50, 19.85]). On the other hand, our analysis does not show any time effects for shoulder girdle injuries, shoulder injuries, forearm fractures, wrist fractures, and hand injuries (Figure 8).

## Discussion

Our study describes the epidemiology of ski/snowboarding-related upper extremity injuries, confirming the hypothesis that carve skiing is a risk factor for upper extremity injuries, as it has already been proven to be for the knee.^8,9^

Alpine skiing represents the most popular winter sport, with for example up to one-third of the population practicing in some countries such as Switzerland.^87^ The most commonly injured body part in adult skiers is the knee. Thus, most of the published articles addressing musculoskeletal injuries in winter sports focus on knees or lower limb pathology in alpine skiing. Regarding upper extremities, the most common injuries are a skier's thumb for skiing^88^ and wrist fractures for snowboarding.^27,89^ Injuries to other upper limb locations have been less frequently analyzed.

In both skiing and snowboarding, injuries were located far from the elbow most frequently (37% of upper extremity injuries to the shoulder and 18% to the hand in skiing, 35% to the wrist, and 27% to the shoulder in snowboarding).

The upper extremity injury distribution differed between the two sports, with the incidence of fractures being significantly higher in snowboarding for the shoulder girdle, humeral diaphysis, fractures around the elbow, forearm, and wrist fractures. In contrast, proximal humerus and hand fractures were more likely to appear while skiing. Our analysis confirms that skiing is more frequently associated with hand injuries, likely due to ski pole use, and with proximal humerus fractures. The latter may be attributed to the higher age distribution of some skiers, as older individuals are more prone to fragility fractures. The incidence of dislocations was significantly higher in snowboarding for the shoulder girdle, shoulder, and elbow. Telemark was associated with shoulder and hand injuries, with incidence rates that were comparable to skiing.

To address the question of the impact of carving on upper extremity injuries, we performed a time-based analysis by creating four groups (before year 2000, 2000 to 2005, 2005 to 2010, and after 2010) with the median year of each data set being used as a new variable for a standardized normal distribution. Our results confirmed the hypothesis that carve skiing introduction is associated with an increased incidence of most upper extremity traumatic conditions. In fact, over time, all segments (except the elbow, where data is lacking) have shown a significant increase in fracture rate. In addition, both shoulder girdle dislocations and hand sprains also had an increase in their incidence with carving.

Since fractures need a certain amount of energy to occur, this change in epidemiology could be explained by the higher kinetics associated with carve skiing accidents. Carving skis are designed to be easier to turn and lead to less drifting while turning. This design means less energy is lost in friction with the snow, potentially resulting in higher downhill speeds. The implications for equipment producers are significant. Manufacturers may need to reconsider the design and safety features of carving skis. For instance, incorporating materials or technologies that enhance stability and control at high speeds could help mitigate the risk of high-energy impacts and subsequent fractures. Additionally, producers might invest in research and development to create skis that balance performance with enhanced safety features, such as improved edge grip without sacrificing speed or maneuverability. By addressing these factors, ski equipment manufacturers can play a crucial role in reducing injury rates and improving the overall safety of the sport, while still catering to the demand for high-performance skiing gear.

Another hypothesis that could explain these trends is the larger edge angle of carving skis, which allows for sharper turns and more aggressive skiing techniques.

The edge angle is the angle the skier makes with the slope during the turn, almost laying inside the bend; the bigger this is, the closer the skier's upper body is to the ground. The upper extremity is therefore more at risk of getting injured in case of a fall. This could especially explain the increase in hand fractures and sprains.

Another factor to keep in mind is that with material that makes it easier to ski, more people with less skiing experience are tempted to try this sport out. Between 2008 and 2014, the rate of active skiers went up 8.8% in Switzerland^83^ (compared to snowboarding which stayed at the same rate).

Initially dominated by younger individuals, snowboarding gained popularity in the 1990s, leading to a broader age distribution over time. However, in contrast to skiing, snowboarding did not go through a big change in technology that drastically modified the technique in the last two decades, and this could explain why upper extremity injuries remained the same during this time period, except for the incidence of elbow fractures that increased. In comparison to skiing, snowboarding did not go through a big change in technology that drastically modified the technique in the last two decades, and this could explain why upper extremity injuries remained the same during this time period. This further strengthens our hypothesis, that only a big change in technology could have an impact on epidemiology.

The limitations of our review are primarily related to the heterogeneity found between the included studies. Both anatomical locations and injury types are inconsistently reported, which could lead to inaccurate or overlapping information. This heterogeneity poses several challenges. Some studies use joint-based classifications, while others use segment-based classifications, leading to potential discrepancies in data interpretation. Additionally, differences in data collection methods could introduce reporting bias, as some studies may have relied on hospital data, while others conducted direct surveillance on the slopes, potentially leading to variations in injury incidence reporting.

Studies conducted in different geographic regions may reflect varying skiing practices, environmental conditions, and healthcare systems, which can further contribute to the heterogeneity of the data. The sample sizes and demographic characteristics (such as age, sex, and skill level of skiers) of the populations studied may also differ significantly between studies, influencing the generalizability of the findings. We did not explicitly compare the demographics between skiers and snowboarders, which could provide further insights into how these factors influence injury patterns.

Changes in skiing equipment technology and techniques over time are not always accounted for consistently in the studies. This inconsistency makes it difficult to isolate the impact of specific factors. As equipment evolves, so do injury patterns, and studies conducted at different times may reflect these changes differently.

Furthermore, we did not explicitly assess potential confounders such as improvements in diagnostic capabilities, an increase in beginner skiers, changes in drug or alcohol consumption, or shifts in skier demographics, which could have influenced the trends observed in injury patterns. Finally, it is possible that some minor sprains were either misclassified or not reported at all, leading to potential underestimation of certain injury types.

The strength of our study is the great number of patients that were included thanks to a systematic literature search using the PRISMA process.

Our study could help improve segment-specific protective equipment to prevent specific lesions in the most affected segments.

## Conclusion

This review confirms that snowboarding is more dangerous for upper extremities with a 10% higher rate of injury compared to skiing. Focusing on the fractures, they are also more frequent among snowboarders with two exceptions: proximal humerus and hand fractures. Finally, our study also confirms that carving is not only a risk factor for knee injuries but also for upper extremity injuries. As for upper extremity injuries, as to our question; to ski or not to ski? Skiing has proven to be the safer sport and will take the podium on this one.
